# Multi-omics profiling-derived signature links cellular ecosystem to glioblastoma prognosis

**DOI:** 10.1016/j.isci.2026.115982

**Published:** 2026-05-18

**Authors:** Zhen Zhang, Hao Xu, Haijing Zheng, Zhaolong Pan, Mei Feng, Yongchang Yang, Manqing Cao

**Affiliations:** 1 Department of Neuro-Oncology and Neurosurgery, Tianjin Medical University Cancer Institute & Hospital, National Clinical Research Center for Cancer, Key Laboratory of Cancer Prevention and Therapy, Tianjin’s Clinical Research Center for Cancer, Tianjin 300060, China; 2 The Second Surgical Department of Breast Cancer, Tianjin Medical University Cancer Institute & Hospital, National Clinical Research Center for Cancer, Tianjin’s Clinical Research Center for Cancer, Key Laboratory of Breast Cancer Prevention and Therapy, Tianjin Medical University, Ministry of Education, Key Laboratory of Cancer Prevention and Therapy, Tianjin 300060, China; 3 Department of Hepatobiliary Cancer, Research Center for Prevention and Treatment of Liver Cancer, Tianjin Medical University Cancer Institute & Hospital, National Clinical Research Center for Cancer, Tianjin’s Clinical Research Center for Cancer, Tianjin Key Laboratory of Digestive Cancer, Tianjin, China; 4 Division of General Surgery, Peking University First Hospital, Peking University, No. 8 Xi Shiku Street, Beijing 100034, China; 5 Department of Radiation Oncology, The First Affiliated Hospital of Guangxi Medical University, Nanning, Guangxi 530022, China

**Keywords:** Disease, Oncology, Transcriptomics

## Abstract

Glioblastoma (GBM) remains a devastating brain malignancy with a dismal prognosis, underscoring the urgent need for robust prognostic biomarkers and therapeutic targets. Here, we developed and validated a seven-gene extracellular matrix-related prognostic signature (ECMSig) using multi-omics data. The ECMSig robustly stratified GBM patients into high- and low-risk groups with distinct overall survival in The Cancer Genome Atlas cohort and Chinese Glioma Genome Atlas cohorts. High ECMSig scores were associated with aggressive molecular features, including upregulation of epithelial-mesenchymal transition and hypoxia, and a tumor-promoting immune microenvironment. Single-cell RNA sequencing analysis identified prognostic Scissor-Positive tumor, myeloid, and endothelial cells exhibiting high ECMSig scores, mesenchymal/immunosuppressive phenotypes, and notable metabolic reprogramming. These cells orchestrate a complex intercellular communication network and spatially co-localize within hypoxic perivascular niches. Furthermore, ECMSig predicted differential drug sensitivities, offering potential therapeutic avenues. The prognostic ECMSig highlights the complex interplay within the GBM ecosystem, paving the way for personalized therapeutic strategies.

## Highlights

• A seven-gene extracellular matrix signature predicts glioblastoma survival
• High-risk glioblastoma shows aggressive and immunosuppressive microenvironment
• High-risk glioblastoma cells co-localized in the hypoxic perivascular niches
• The gene signature predicts drug sensitivities for personalized therapy

## Graphical abstract

**Figure:**
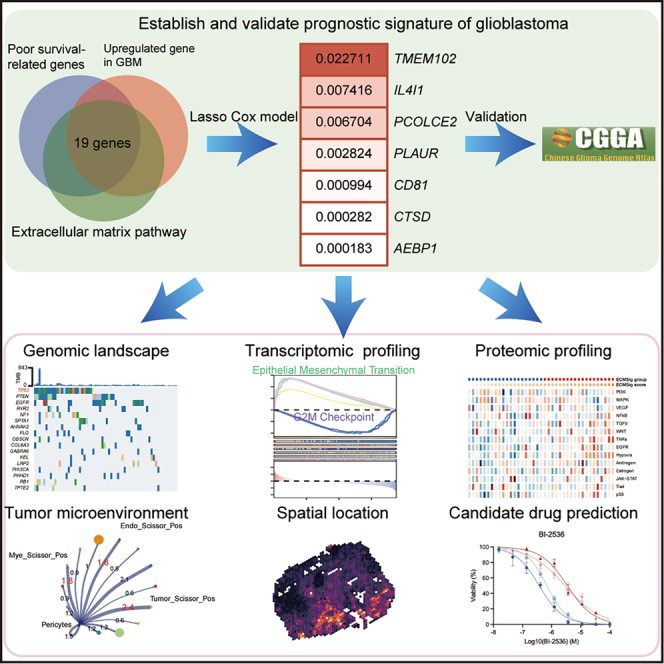

## Introduction

Glioblastoma (GBM) is the most prevalent and aggressive primary malignant brain tumor in adults, characterized by rapid cellular proliferation, diffuse infiltration into the brain parenchyma, and profound therapeutic resistance.^1^ Despite multimodal treatment strategies encompassing maximal safe surgical resection followed by radiotherapy and concomitant/adjuvant temozolomide chemotherapy, the median overall survival (OS) for GBM patients remains distressingly short, typically around 15–17 months, with a 5-year survival rate at 4.7%.^1^^,^^2^ This grim outlook highlights the critical unmet need for more accurate prognostic stratification beyond conventional clinical factors and established molecular markers like *MGMT* promoter methylation,^3^ as well as for the identification of therapeutic targets to improve patient outcomes.

The GBM tumor microenvironment (TME) is an intricate and dynamic ecosystem comprising not only cancer cells but also a diverse array of non-malignant cells, including immune cells, stromal cells, endothelial cells forming the tumor vasculature, and an abundant, extensively remodeled extracellular matrix (ECM).^4^^,^^5^ The ECM, once viewed merely as a structural scaffold, is now recognized as a critical bioactive component that profoundly influences virtually all aspects of cancer biology.^6^ In GBM, the ECM contributes to tumor growth, invasion, angiogenesis, stemness, immune modulation, and resistance to therapy.^7^^,^^8^ Dysregulation of ECM components, such as collagens, proteoglycans, matricellular proteins, and ECM-remodeling enzymes, is a hallmark of GBM, creating a permissive niche for tumor progression.^9^^,^^10^

Given the pivotal role of the ECM in GBM pathogenesis, ECM-related genes and pathways represent a promising source for prognostic biomarker development. While individual ECM components have been linked to GBM prognosis, the inherent complexity and heterogeneity of GBM suggest that multi-gene signatures integrating information from several ECM-related players might offer superior prognostic accuracy and biological insight compared to single markers. Several gene expression-based signatures have been developed for GBM, focusing on various biological aspects like mesenchymal transition, immune response, or stemness.^11^^,^^12^ However, a comprehensive prognostic signature specifically derived from and focused on the broad spectrum of ECM-related genes, and subsequently validated across multiple omics layers and resolutions, remains an area of active investigation.

Therefore, this study aimed to develop and rigorously validate an ECM-related gene signature (ECMSig) for GBM prognosis. We hypothesized that such a signature could not only accurately predict patient survival but also unveil key biological mechanisms and cellular interactions within the ECM-rich TME that drive GBM aggressiveness. To achieve this, we first constructed the ECMSig using transcriptomic data from TCGA-GBM cohort by integrating differentially expressed genes (DEGs), prognosis-associated genes, and ECM pathway information. The signature’s prognostic robustness was then validated in two large Chinese Glioma Genome Atlas (CGGA) cohorts. We further investigated the genomic, transcriptomic, and proteomic alterations associated with ECMSig stratification, dissecting its links to oncogenic signaling, hypoxia, and immune infiltration. Leveraging single-cell RNA sequencing (scRNA-seq) data, we identified specific prognostically detrimental cell subpopulations characterized by high ECMSig scores and explored their functional states, metabolic reprogramming, and intercellular communication networks. Finally, using spatial transcriptomics, we examined the spatial organization of these features within the tumor architecture and explored the therapeutic implications of ECMSig by predicting drug sensitivities and *in vitro* functional assays. Our comprehensive approach seeks to establish the ECMSig as a valuable prognostic tool and provide deeper insights into the ECM-mediated pathobiology of GBM.

## Results

### Development and validation of an ECM-related prognostic signature in GBM

**Figure 1:**
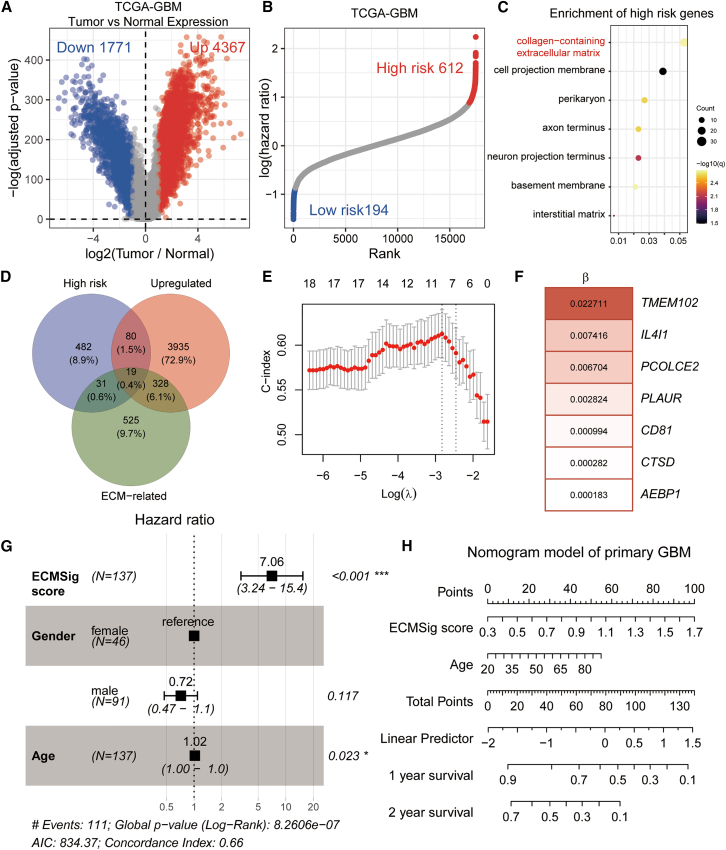
Development and validation of an ECMSig in GBM (A) Volcano plot of DEGs between GBM tumor tissues and normal brain tissues from TCGA-GBM cohort. Red dots represent 4,367 upregulated genes, and blue dots represent 1,771 downregulated genes in tumors. Benjamini-Hochberg adjusted. (B) Ranked distribution of log(hazard ratios) from univariate Cox regression analysis for all expressed genes in the TCGA-GBM cohort. Red dots indicate 612 genes associated with poor prognosis (*p* < 0.05), and blue dots indicate 194 genes associated with favorable prognosis (*p* < 0.05). Log-rank test. (C) Gene ontology (GO) enrichment analysis of genes associated with poor patient OS in GBM cohort. Dot plot displaying the top enriched GO biological process terms. The x axis represents the gene ratio. The size of the dots corresponds to the number of genes associated with each term, and the color intensity reflects the statistical significance (-log10[q value]). Benjamini-Hochberg adjusted. (D) Venn diagram illustrating the intersection of genes upregulated in GBM tumors, genes associated with high risk (poor prognosis) in the TCGA-GBM cohort, and ECM-related genes, yielding 19 candidate genes for signature construction. Numbers in overlaps represent gene counts. (E) 10-fold cross-validation for tuning parameter (λ) selection in the LASSO Cox model. Red dots represent partial likelihood deviance values, and error bars indicate standard errors. The vertical dotted lines indicate the optimal λ value (left) that gives the best C index and the λ value corresponding to one standard error (right). (F) The seven genes included in the final ECMSig and their corresponding LASSO coefficients. Red bars indicate positive coefficients (associated with higher risk). (G) Forest plot of multivariate Cox regression analysis for OS in the TCGA-GBM cohort (*n* = 137). Hazard ratios (HRs) with 95% confidence intervals (CIs) and *p* values are shown. ∗∗∗*p* < 0.001, ∗*p* < 0.05. (H) Nomogram for predicting 1-year and 2-year OS probabilities in GBM patients, incorporating the ECMSig score and age. Points are assigned for each variable, summed to a total score, which corresponds to predicted survival probabilities. See also Figure S1.

To identify genes associated with GBM tumorigenesis and prognosis, we first performed differential expression analysis between tumor and normal tissues using the TCGA-GBM cohort. This analysis revealed 4,367 upregulated and 1,771 downregulated genes in GBM tumors compared to normal tissues (Figure 1A; adjusted *p* value <0.05, |log_2_(fold change)| > 1). Pathway enrichment analysis showed that “focal adhesion,” “cell cycle checkpoints,” and “collagen-containing ECM” pathways were upregulated in primary GBM tumor samples, and “neuronal system,” “synaptic membrane,” and “neuron to neuron synapse” pathways were downregulated in tumor (Figure S1A). Subsequently, univariate Cox proportional hazards regression analysis was performed on the same cohort to identify prognosis-related genes. We identified 612 genes significantly associated with poor prognosis (higher hazard ratio, *p* < 0.05) and 194 genes associated with favorable prognosis (lower hazard ratio, *p* < 0.05) (Figure 1B). Pathway enrichment analysis showed that poor-prognosis-associated genes were enriched in “collagen-containing ECM” and “cell projection membrane” pathways (Figure 1C), but superior-prognosis-associated genes were enriched in “eukaryotic translation initiation” pathway (Figure S1B). Remarkably, both upregulated genes and poor prognosis-related genes showed significant enrichment in ECM-related pathways. This concordance suggests that ECM components are involved not only in GBM tumorigenesis but also in determining clinical outcomes. Therefore, we intersected the two gene sets with ECM pathway-annotated genes, yielding 19 candidate genes (Figure 1D). This strategy ensured that the final candidates were biologically relevant to ECM remodeling and clinically relevant to patient survival, thereby providing a strong basis for constructing a prognostic ECM signature.

Using these 19 candidate genes, we employed least absolute shrinkage and selection operator (LASSO) Cox regression analysis in the TCGA-GBM training cohort to develop a prognostic signature. The LASSO coefficient profiles for the candidate genes across different log(λ) values are shown in Figure S1C. 10-fold cross-validation was used to determine the optimal λ value, which resulted in a model with the best C index (Figure 1E). This process led to the construction of a seven-gene ECM-related prognostic signature (ECMSig), consisting of *TMEM102*, *IL4I1*, *PCOLCE2*, *PLAUR*, *CD81*, *CTSD*, and *AEBP1* (Figure 1F). The risk score for each patient was calculated as a linear combination of the expression levels of these seven genes, weighted by their respective LASSO coefficients.

To assess the independent prognostic value of the ECMSig, we performed multivariate Cox regression analysis including the risk score, patient age, and gender in the TCGA-GBM cohort (*n* = 137). The ECMSig score emerged as a significant and independent predictor of OS (HR = 7.06, 95% CI = 3.24–15.4, *p* < 0.001) (Figure 1G). Furthermore, a nomogram integrating the ECMSig score, age, and gender was constructed to provide a quantitative tool for predicting 1-year and 2-year survival probabilities for GBM patients (Figure 1H).

### External validation of the prognostic value of ECMSig score

To further evaluate the prognostic robustness of the seven-gene ECMSig, we validated its performance in two independent external cohorts: CGGA_325 (*n* = 325) and CGGA_693 (*n* = 693). Primary GBM patients in each cohort were stratified into high-risk and low-risk groups based on the median ECMSig score calculated using the same formula derived from the TCGA-GBM training set.

**Figure 2:**
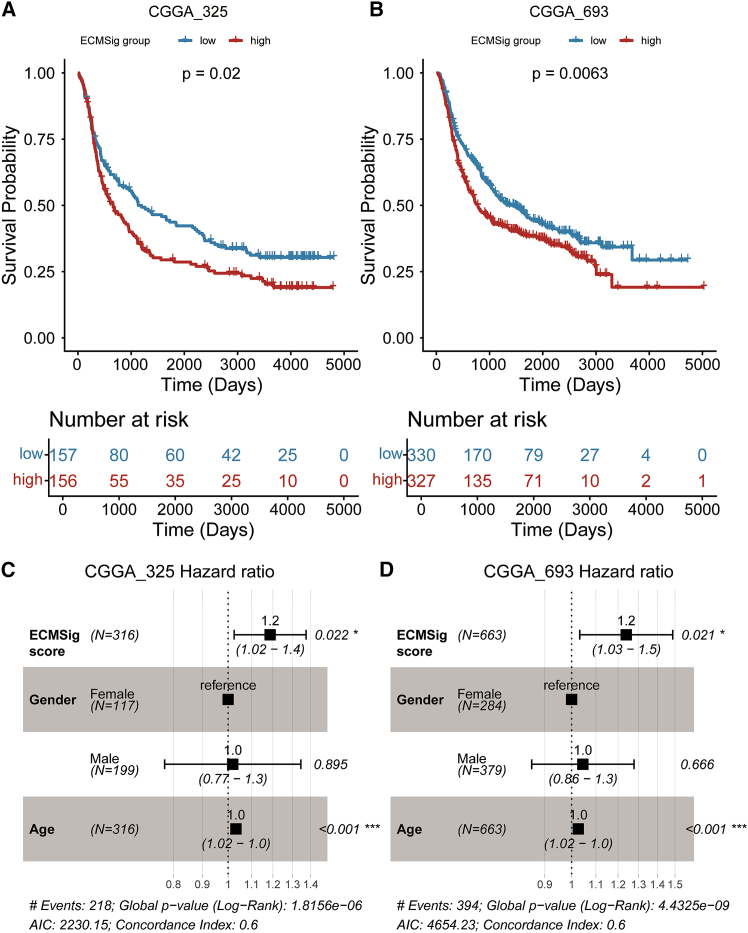
Validation of the ECMSig in independent CGGA cohorts (A and B) Kaplan-Meier survival curves for OS of patients stratified by the ECMSig score (high-risk vs. low-risk based on median score) in (A) the CGGA_325 cohort and (B) the CGGA_693 cohort. *p* values were calculated using the log-rank test. (C and D) Forest plots of multivariate Cox regression analysis for OS in (C) the CGGA_325 cohort and (D) the CGGA_693 cohort (*n* = 663 with complete data), including the ECMSig score, gender, and age. Hazard ratios (HRs) with 95% confidence intervals (CIs) and *p* values are shown. Global *p* value, AIC, and Concordance Index for the multivariate models are also provided. ∗∗*p* < 0.01, ∗∗∗*p* < 0.001. See also Figure S2.

In the CGGA_325 cohort, Kaplan-Meier survival analysis revealed that patients in the high-risk group had significantly shorter OS compared to those in the low-risk group (Figure 2A; log-rank *p* = 0.02). Similarly, in the larger CGGA_693 cohort, the ECMSig effectively discriminated between patients with poor and favorable prognosis, with the high-risk group exhibiting significantly worse OS (Figure 2B; log-rank *p* = 0.0063). To ascertain whether the ECMSig retained its prognostic significance independently of established clinical factors, we performed multivariate Cox regression analyses in both validation cohorts. In the CGGA_325 cohort, after adjusting for patient age and gender, the ECMSig score remained a significant independent prognostic factor for OS (HR = 1.2, *p* = 0.022; Figure 2C). Consistent results were observed in the CGGA_693 cohort, where the ECMSig score also demonstrated independent prognostic value (HR = 1.2, *p* = 0.021; Figure 2D). These findings across multiple independent datasets underscore the generalizability and reliability of the ECMSig as a prognostic tool for GBM patients.

To examine the pan-cancer relevance of ECMSig, we extended the prognostic analysis to all TCGA tumor types. ECMSig was significantly associated with OS in low-grade glioma (*p* = 0.00033, Figure S2A), lung squamous cell carcinoma (LUSC, *p* = 0.029, Figure S2B), and cervical squamous cell carcinoma/endocervical adenocarcinoma (*p* = 0.023, Figure S2C), but not in other cancer types after multiple-testing correction. These results suggest that although the signature captures ECM remodeling processes that may influence survival in certain contexts, its strongest and most consistent prognostic impact is observed in GBM.

### Genomic alterations associated with the ECMSig in GBM

**Figure 3:**
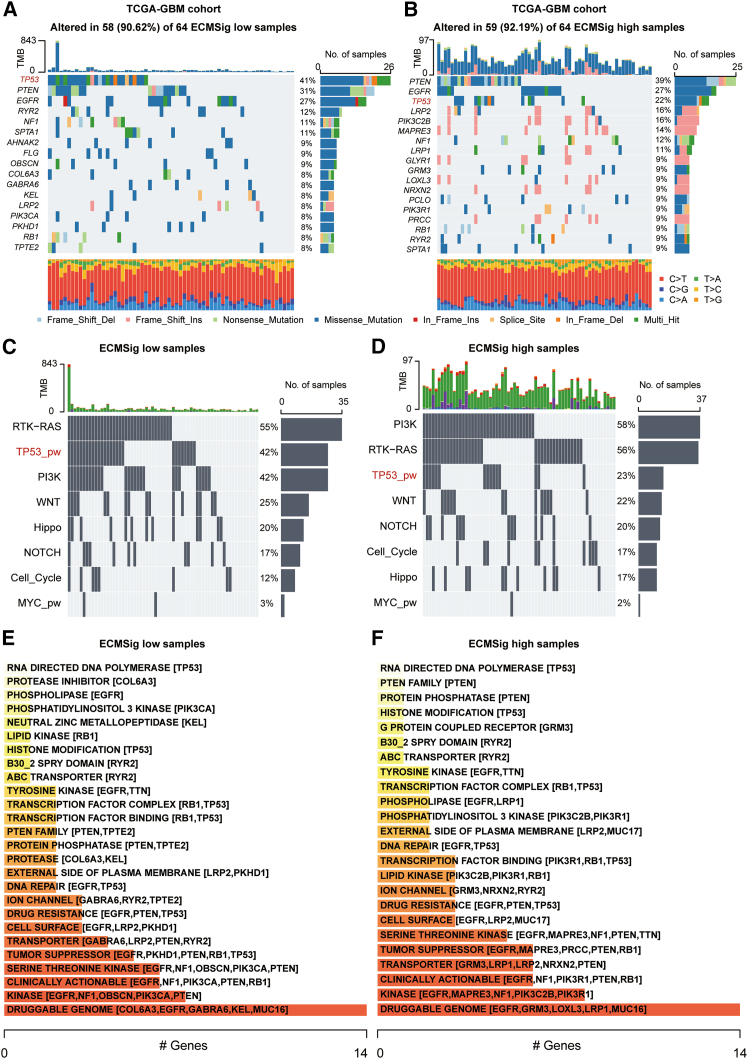
Genomic alterations in ECMSig-low and ECMSig-high GBM groups from TCGA (A and B) Oncoprints displaying somatic mutations in frequently altered genes for (A) 64 ECMSig-low samples and (B) 64 ECMSig-high samples. Genes are listed on the left, with mutation frequencies (%) on the right. Mutation types are color-coded at the bottom. Each column represents a sample. (C and D) Bar plots showing the percentage of samples with mutations in key signaling pathways for (C) ECMSig-low samples and (D) ECMSig-high samples. (E and F) Drug-gene interaction summaries for (E) ECMSig-low samples and (F) ECMSig-high samples, categorized by druggable pathways/mechanisms. Red bars highlight clinically actionable genes with the number of affected genes indicated. See also Figure S3.

To explore the genomic landscape underlying the prognostic stratification defined by the ECMSig, 128 TCGA-GBM patients with matched genomic sequencing data and bulk RNA-seq data were stratified into TCGA-GBM ECMSig-low (64 samples) and TCGA-GBM ECMSig-high (64 samples) risk groups. Oncoprint plots illustrating the distribution of somatic mutations in frequently altered genes for 64 ECMSig-low samples and 64 ECMSig-high samples are presented (Figures 3A and 3B). Among the most frequently mutated genes in the ECMSig-low group were *TP53* (41%), *PTEN* (31%), *EGFR* (27%), *RYR2* (12%), and *NF1* (11%) (Figure 3A).

While many frequently mutated genes were common to both groups, notable differences in mutation frequencies were observed in the ECMSig-high group. For instance, *TP53* mutations were significantly more prevalent in the ECMSig-low group (41%) compared to the ECMSig-high group (22%, *p* = 0.035, Figure S3A). Other frequently mutated genes in the high-risk group included *PTEN* (39%), *EGFR* (27%), *NF1* (12%), and *RB1* (9%).

We next investigated the differential landscape of mutated signaling pathways between the two risk groups. In the ECMSig-low group, pathways frequently affected by somatic mutations included RTK-RAS (55%), TP53 (42%), PI3K (42%), WNT (25%), Hippo (20%), Notch (17%), and cell cycle (12%) (Figure 3C). In the ECMSig-high group, the mutational landscape of these pathways showed some shifts: PI3K (58%), RTK-RAS (56%), TP53 (23%), WNT (22%), Notch (20%), Hippo (17%), and cell cycle (17%) (Figure 3D). The most striking difference was the significantly lower frequency of *TP53* pathway mutations in the ECMSig-high group (Figure S3B), consistent with the gene-level observation. Conversely, PI3K pathway alterations appeared more frequent in the ECMSig-high group.

To assess potential therapeutic vulnerabilities linked to these genomic alterations, we analyzed druggable gene interactions in both groups. In the ECMSig-low group, a panel of druggable categories was identified, including DNA repair (*TP53*) and tyrosine kinase signaling (*EGFR*) (Figure 3E). Similarly, the ECMSig-high group presented druggable targets, with notable representation in PI3K signaling and receptor tyrosine kinases (Figure 3F). Drug-gene interaction analysis also showed that therapies targeting wild-type p53 mechanisms or specific components of the PI3K pathway might have differential efficacy depending on the ECMSig risk group. The co-occurrence and mutual exclusivity of different mutations have also been evaluated (Figure S3C). The significant co-occurrence of driver mutations, such as *RB1* and *TP53*, suggests a potential drug combination during treatment of GBM patients in different ECMSig groups. Taken together, these results suggest that two ECMSig groups exhibit distinct genomic landscape, which might result in different TME and therapeutic strategies.

### Transcriptomic and immune microenvironment features of ECMSig-stratified GBM

**Figure 4:**
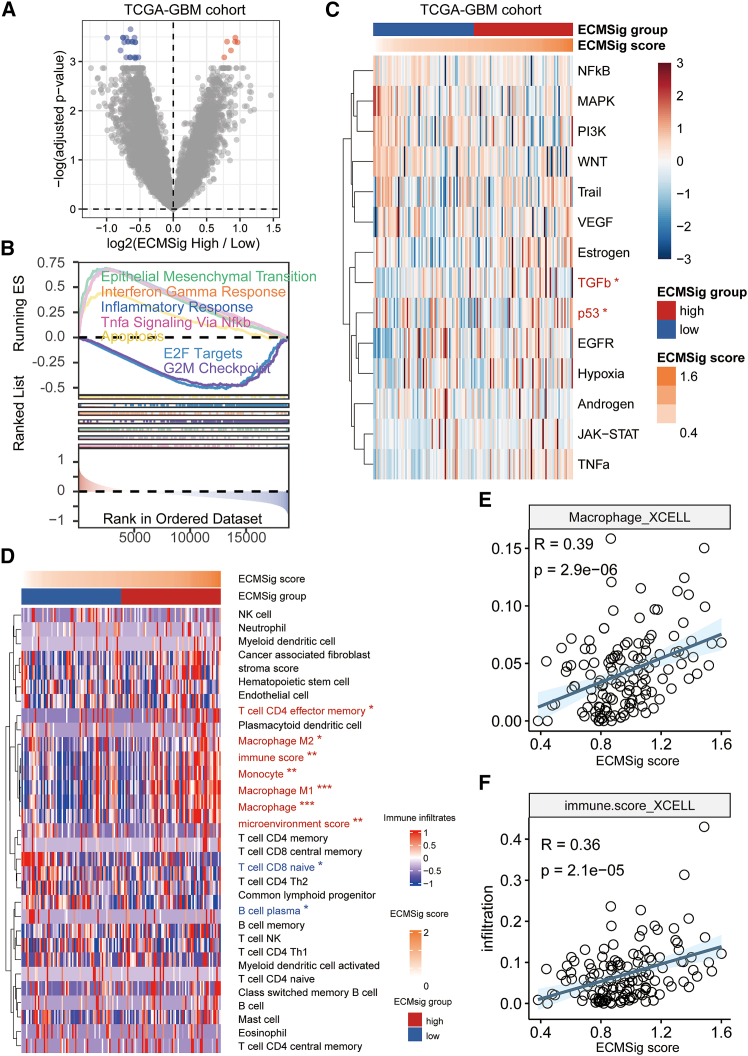
Transcriptomic and TME characteristics associated with ECMSig in TCGA-GBM cohort (A) Volcano plot showing DEGs between ECMSig-high and ECMSig-low groups. Red dots: upregulated in high-risk; blue dots: upregulated in low-risk. Benjamini-Hochberg adjusted. (B) Gene set enrichment analysis (GSEA) plots showing enrichment of hallmark pathways. Pathways enriched in ECMSig-high and ECMSig-low groups are shown with their running enrichment scores (ESs) and ranked gene lists. Benjamini-Hochberg adjusted. (C) Heatmap showing the activity scores of selected oncogenic and tumor-related signaling pathways (rows) across TCGA-GBM samples (columns), annotated by ECMSig group and ECMSig score. Red indicates high activity, blue indicates low activity. ∗*p* < 0.05. Wilcoxon signed-rank test. (D) Heatmap depicting the estimated infiltration levels of various immune and stromal cell types (rows) in TCGA-GBM samples (columns), stratified by ECMSig group and score. Red indicates high infiltration, blue indicates low infiltration. Cells significantly highly infiltrated in ECMSig-high are labeled in red, and those high in ECMSig-low group are in blue. ∗q < 0.05, ∗∗q < 0.01, ∗∗∗q < 0.001. Wilcoxon signed-rank test. Benjamini-Hochberg adjusted. (E and F) Scatterplots showing the spearman correlation between ECMSig score and (E) Macrophage_XCELL infiltration score and (F) immune_score_XCELL. The blue line represents the linear regression fit with 95% confidence interval bands. Spearman correlation test.

To further elucidate the biological processes underpinning the prognostic power of the ECMSig, we performed a comprehensive transcriptomic analysis comparing ECMSig-high and ECMSig-low risk groups in the TCGA-GBM cohort. Differential gene expression analysis revealed numerous genes significantly dysregulated (Figure 4A). Gene set enrichment analysis (GSEA) was then conducted to identify pathways differentially enriched between two groups (Figure 4B). Notably, the ECMSig-high group exhibited significant enrichment of pathways associated with adverse prognosis and aggressive tumor biology. These included epithelial-mesenchymal transition (EMT, a key driver of tumor invasion and metastasis), inflammatory response, and signaling pathways such as TNFα Signaling via NF-κB and apoptosis. Notably, cell cycle-related E2F targets and G2M checkpoint pathway were significantly enriched in the ECMSig low group. Activities of 14 oncogenic and tumor-related signaling pathways were also assessed (Figure 4C). The heatmap visualization demonstrates that ECMSig-high and ECMSig-low tumors were characterized by distinct activity of these pathways, such as p53 pathway. These results suggest distinct transcriptional programs are active in these prognostically divergent patient subsets.

Given the enrichment of inflammatory pathways in ECMSig-high tumors, and the known importance of the TME in GBM, we next investigated the immune cell infiltration landscape using xCell (Figure 4D). The heatmap displays the relative abundance of various immune and stromal cell populations. ECMSig-high tumors were characterized by a distinct immune signature, with notably higher estimated infiltration of M2 macrophages, known to promote tumor progression and immunosuppression. Conversely, ECMSig-low tumors showed higher infiltration of CD8+ T cell and B/Plasma cells.

Specifically, there was a significant positive correlation between the ECMSig score and the infiltration level of macrophages, as estimated by xCell (Spearman R = 0.39, *p* = 2.9e−06) (Figure 4E). Furthermore, the overall immune infiltration score, reflecting the combined activity of various stromal and immune components, also showed a positive correlation with the ECMSig score (Spearman R = 0.36, *p* = 2.1e−05) (Figure 4F). These results strongly suggest that the poor prognosis associated with a high ECMSig score is, at least in part, mediated by a tumor-promoting and immunosuppressive TME, with prominent roles for macrophage infiltration and hypoxia.

### Proteomic validation of ECMSig-associated features in an independent GBM cohort

**Figure 5:**
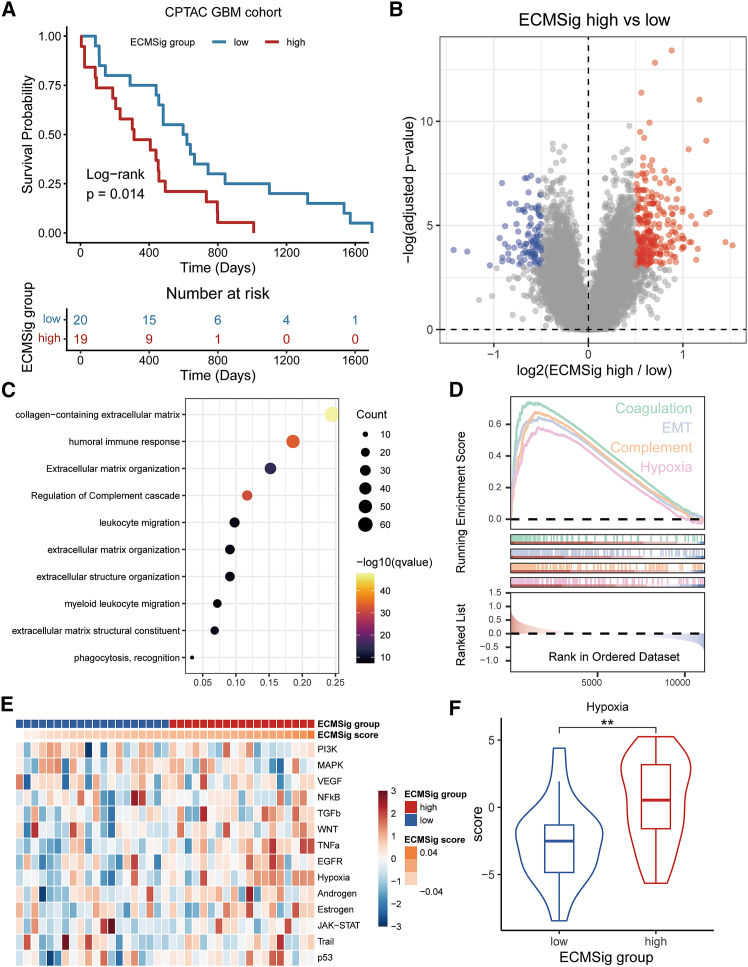
Proteomic validation of ECMSig-associated features in the CPTAC GBM cohort (A) Kaplan-Meier survival curve for OS of CPTAC GBM patients (*n* = 39; low-risk *n* = 20, high-risk *n* = 19) stratified by the ECMSig score (protein level). *p* value from log-rank test. (B) Volcano plot of differentially abundant proteins between ECMSig-high and ECMSig-low groups in the CPTAC cohort. Red dots: higher abundance in high-risk; blue dots: higher abundance in low-risk. (C) Dot plot of Gene Ontology (GO) and pathway enrichment analysis for proteins upregulated in the ECMSig-high group. Dot size represents gene count; color intensity represents –log10(q value). Benjamini-Hochberg adjusted. (D) GSEA plots showing enrichment of hallmark pathways at the protein level in the ECMSig-high group. Benjamini-Hochberg adjusted. (E) Heatmap showing the activity scores of selected signaling pathways based on protein abundance across CPTAC samples (columns), stratified by ECMSig group and score. (F) Violin plot comparing the hypoxia signature score (protein level) between ECMSig-low and ECMSig-high groups. ∗∗*p* < 0.01. Wilcoxon signed-rank test.

To validate our transcriptomic findings at the protein level and in an independent patient cohort, we utilized data from the Clinical Proteomic Tumor Analysis Consortium (CPTAC) GBM confirmatory cohort. First, we assessed the prognostic performance of our transcriptomically derived ECMSig in this proteomic dataset. All seven ECMSig genes were successfully detected at protein level, and patients were stratified into ECMSig-high and ECMSig-low groups by the ECMSig score (detailed in STAR Methods). Kaplan-Meier analysis demonstrated that patients in the ECMSig-high group had significantly poorer OS compared to those in the ECMSig-low group (Figure 5A; log-rank *p* = 0.014), thereby confirming the prognostic relevance of the ECMSig at the proteome level.

Next, we performed differential protein expression analysis between the ECMSig-high and ECMSig-low groups within the CPTAC cohort (Figure 5B). Functional enrichment analysis of the differentially abundant proteins (Figures 5C and 5D) revealed that proteins upregulated in the ECMSig-high group were significantly enriched in pathways and biological processes highly relevant to ECM biology and hypoxia, underscoring the strong association of the ECMSig-high proteome with ECM remodeling and immune modulation.

Additionally, a heatmap visualizing the activity of key oncogenic and tumor-related signaling pathways based on protein abundance (Figure 5E) mirrored the patterns observed in the transcriptome analysis. ECMSig-high tumors showed elevated protein-level activity in hypoxia and TNFα pathways. Notably, the hypoxia score was significantly higher in the ECMSig-high group compared to the ECMSig-low group (Figure 5F), providing strong proteomic evidence for an association between high ECMSig status and increased tumor hypoxia. These proteomic analyses robustly validate the key transcriptomic features associated with the ECMSig, particularly the link to ECM dysregulation and a hypoxic TME.

### Single-cell resolution of ECMSig expression and identification of prognostically relevant cellular states in GBM

**Figure 6:**
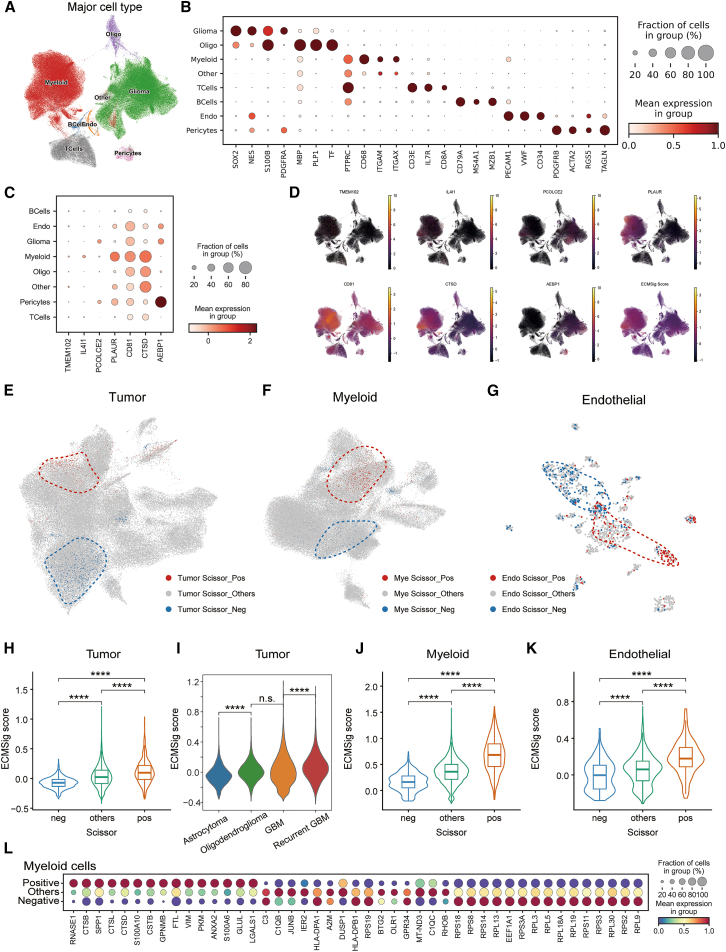
Single-cell RNA sequencing analysis revealing ECMSig expression across cell types and identification of prognostically relevant cell states in GBM (A) UMAP visualization of major cell types identified in GBM scRNA-seq data. (B) Dot plot showing the scaled average expression (color intensity) and percentage of cells expressing (dot size) canonical marker genes for each major cell type. (C) Dot plot showing the scaled average expression and percentage of cells expressing the seven ECMSig genes across major cell types. (D) UMAP plots showing the expression levels of individual ECMSig genes and overall ECMSig score across all cells. (E–G) UMAP plots illustrating Scissor-identified prognostically unfavorable (Scissor_Pos, red dashed circle) and favorable (Scissor_Neg, blue dashed circle; Scissor_Others, gray) cell subpopulations within (E) tumor cells, (F) myeloid cells, and (G) endothelial cells. (H–K) Violin plots comparing ECMSig scores among tumor cells grouped by Scissor status (H) and tumor type (I), and myeloid cells (J) or endothelial cells (K) grouped by Scissor status. ∗∗∗∗*p* < 0.0001. Wilcoxon signed-rank test. (L) Dot plot showing differentially expressed marker genes between myeloid Scissor_Pos and other myeloid cells. Dot size indicates the fraction of cells in the group expressing the gene; color indicates average expression level.

To dissect the cellular basis of the ECMSig and identify specific cell populations contributing to its prognostic significance, we analyzed publicly available scRNA-seq data of GBM tumors. Unsupervised clustering and UMAP visualization identified major cell types within the GBM TME, including malignant glioma cells, myeloid cells, oligodendrocytes, T cells, B cells, endothelial cells, and pericytes (Figure 6A). The identity of these clusters was confirmed by the expression of canonical marker genes for each cell type (Figure 6B), such as *SOX2* and *NES* for glioma cells, *PTPRC* (CD45) and *CD68* for myeloid cells, *CD3E* for T cells, and *PECAM1* (CD31) for endothelial cells.

We next examined the expression of the seven ECMSig constituent genes across these identified cell populations (Figure 6C). *CD81* showed broad expression across multiple cell types, including glioma cells, myeloid cells, and endothelial cells, suggesting their roles in intercellular communication and TME modulation. Other genes showed more cell type-specific expression patterns, for instance, *IL4I1* and *AEBP1* were mainly expressed on myeloid cells and pericytes, respectively. UMAP visualizations of individual ECMSig genes further illustrated their distinct and overlapping expression distributions within the TME (Figure 6D), highlighting the complex multicellular (glioma cells, myeloid cells, endothelial cells, and pericytes) contribution to the overall ECMSig score.

Next, we employed the Scissor algorithm to pinpoint cell subsets most strongly associated with patient prognosis.^13^ Scissor is a machine learning-based integrative method that identifies biologically and clinically relevant cell subpopulations within single-cell RNA-seq datasets whose transcriptional profiles are most predictive of a given bulk tissue phenotype, such as worse survival of GBM patients. Positive cells identified by Scissor were determined as the subpopulation that is most associated with worse survival. Notably, Scissor identified prognostically unfavorable subpopulations within tumor cells (tumor Scissor_Pos), myeloid cells (myeloid Scissor_Pos), and endothelial cells (endothelial Scissor_Pos) (Figures 6E–6G; dashed red circles highlight Scissor-Pos cells).

Of note, we investigated the ECMSig score within these prognostically defined cell subpopulations. Compared to their Scissor-negative (Scissor_Neg) counterparts and other cells within the same cell lineage, tumor Scissor_Pos cells exhibited significantly higher ECMSig scores (Figure 6H; *p* < 0.0001). Interestingly, among malignant cell subtypes, recurrent malignant GBM cells exhibited the highest ECMSig scores, suggesting enhanced ECM remodeling activity during recurrence that may contribute to therapeutic resistance and aggressive behavior (Figure 6I). Similarly, myeloid Scissor_Pos cells (Figure 6J; *p* < 0.0001) and endothelial Scissor_Pos cells (Figure 6K; *p* < 0.0001) also displayed significantly elevated ECMSig scores. Further examination of marker genes for myeloid Scissor_Pos cells compared to other myeloid cells revealed the significant upregulation of ECM-related genes including *VIM* (Figure 6L). This indicates that the specific cell states identified by Scissor as being detrimental to patient survival are characterized by high expression of the ECM-related prognostic signature. This observation provides a cellular underpinning for the ECMSig, suggesting that its prognostic power derives from capturing the activity of these pro-malignant cell states within the tumor, myeloid, and endothelial compartments.

### Metabolic reprogramming in prognostically unfavorable Scissor-Positive cell populations

**Figure 7:**
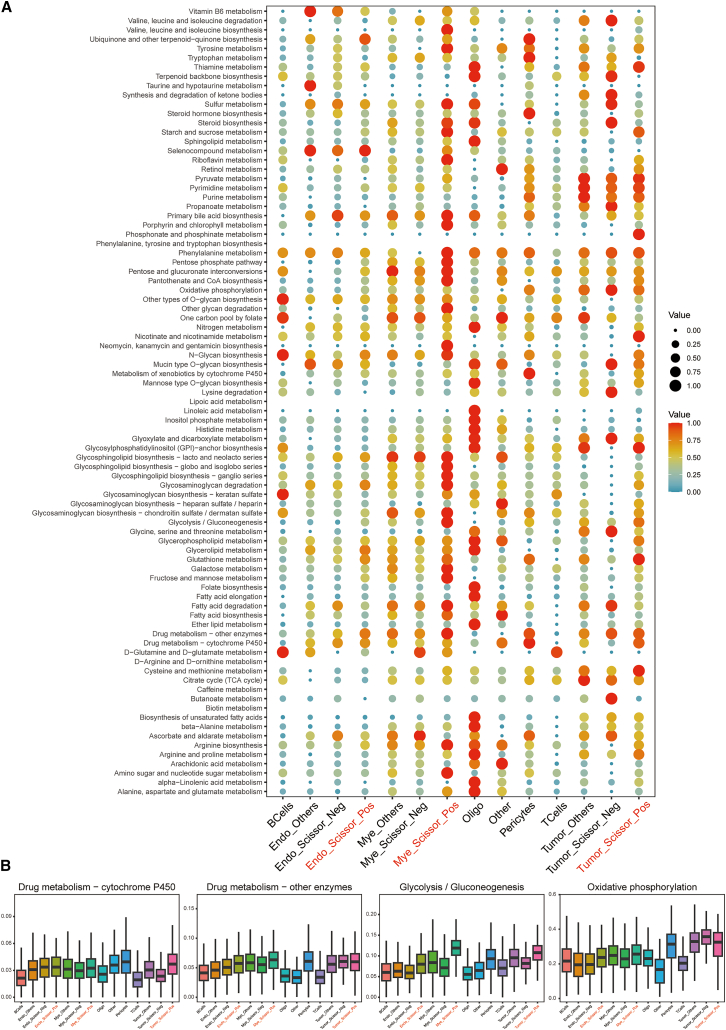
Metabolic characterization of Scissor-identified cell populations in GBM (A) Dot plot heatmap illustrating the activity scores of numerous metabolic pathways (rows) across different cell populations (columns), including major cell types and their Scissor-defined subpopulations. Dot size represents the fraction of cells with the pathway active; color intensity represents the mean activity score. (B) Boxplots comparing the activity scores of selected metabolic pathways across the indicated cell populations.

Given the established link between metabolic reprogramming and cancer progression, particularly in the context of hypoxia and ECM interactions, we utilized scMetabolism to investigate the metabolic features of the Scissor-identified prognostically relevant cell populations. A comprehensive analysis of various metabolic pathways across all identified cell types and Scissor-defined subpopulations revealed distinct metabolic profiles (Figure 7A). Notably, tumor Scissor_Pos, myeloid Scissor_Pos, and endothelial Scissor_Pos cells exhibited heightened activity in several metabolic pathways compared to their Scissor_Neg counterparts or other cells within the same lineage. Myeloid Scissor_Pos cells showed widest metabolic dysregulation compared to other cells. Specifically, tumor Scissor_Pos and myeloid Scissor_Pos cells showed highest activity of glycolysis/gluconeogenesis among all cell types, consistent with the Warburg effect often observed in cancer cells and pro-tumorigenic immune cells adapting to a hypoxic microenvironment (Figure 7B). Furthermore, compared to their counterparts from the same lineage, all three Scissor-Pos cells showed elevated activity of glycolysis. Additionally, these cells also exhibited dysregulated activity of oxidative phosphorylation and drug metabolism, further supporting a shift toward glycolytic metabolism. These findings indicate that the prognostically unfavorable Scissor-Pos cell populations undergo significant metabolic reprogramming, characterized by enhanced glycolysis and altered drug metabolism pathways, likely contributing to their aggressive phenotype and adaptation to the demanding TME. The observed metabolic shift toward hypoxia-associated pathways in these cells further strengthens the link between ECMSig, adverse cell states, and a hypoxic TME.

### Functional states and intercellular communication networks of prognostically detrimental cell subpopulations

**Figure 8:**
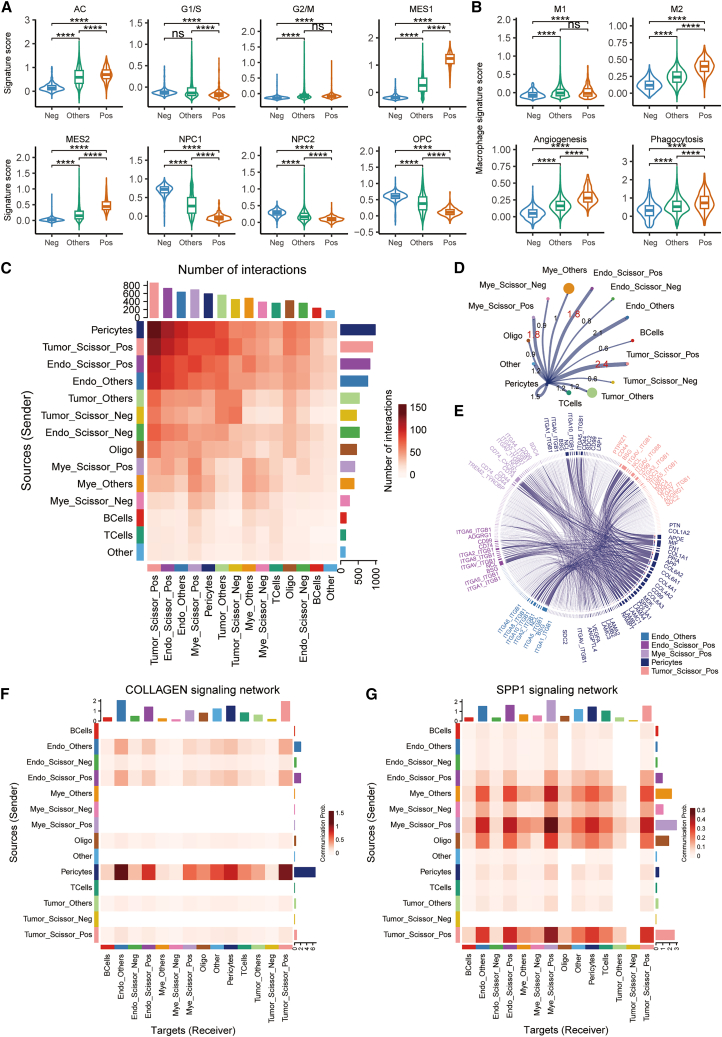
Functional states and intercellular communication networks of prognostically detrimental Scissor-Positive cell subpopulations (A) Violin plots comparing signature scores for glioma cell states among tumor_Scissor_Negative (Neg), tumor_Scissor_Others (Others), and tumor_Scissor_Positive (Pos) cells. ∗∗∗∗*p* < 0.0001, ∗∗∗*p* < 0.001, ∗∗*p* < 0.01, ∗*p* < 0.05, ns = not significant. Wilcoxon signed-rank test. (B) Violin plots comparing signature scores for macrophage polarization (M1, M2) and functions (angiogenesis, phagocytosis) among myeloid_Scissor_Negative (Neg), myeloid_Scissor_Others (Others), and myeloid_Scissor_Positive (Pos) cells. Wilcoxon signed-rank test. (C) Heatmap showing the number of inferred interactions between different cell populations (sources as rows, targets as columns). Color intensity represents the number of interactions. (D) Circle plot visualizing significant intercellular communication pathways. Lines connect interacting cell types, with line thickness/color potentially indicating interaction strength or number of pathways. (E) Chord diagram illustrating specific ligand-receptor pairs mediating interactions between key cell types. (F and G) Heatmaps of inferred communication strength for (F) the collagen signaling network and (G) the SPP1 signaling network. Rows represent sender cell types, columns represent receiver cell types. Color intensity indicates communication probability or strength.

To further characterize the functional roles of the Scissor-Pos cell populations, we examined their expression of established glioma cell state markers^14^ and myeloid cell functional markers.^15^ Within the malignant tumor cell compartment, tumor Scissor_Pos cells exhibited a significant decrease in the neural progenitor cell (NPC)-like state (NPC1 and NPC2 signature scores) and a concomitant significant increase in the mesenchymal (MES)-like state (MES1 and MES2 signature scores) compared to tumor Scissor_Neg and tumor Scissor_Others cells (Figure 8A; *p* < 0.0001). Therefore, the mesenchymal transition in glioma is strongly associated with increased invasiveness, therapeutic resistance, and poor prognosis.

In the myeloid compartment, myeloid Scissor_Pos cells displayed a striking polarization toward an M2-like macrophage phenotype, characterized by significantly higher M2 macrophage signature scores (Figure 8B, *p* < 0.0001) compared to myeloid Scissor_Neg and myeloid Scissor_Others cells. M2-polarized macrophages are known for their immunosuppressive and pro-tumoral functions, promoting angiogenesis, matrix remodeling, and tumor cell invasion.^16^ Accordingly, myeloid Scissor_Pos cells also showed significantly higher scores for angiogenesis (*p* < 0.0001) and phagocytosis (*p* < 0.0001), further highlighting their active role in shaping a tumor-supportive microenvironment.

Given the complex interplay between different cell types in the TME, we next investigated intercellular communication networks using CellChat.^17^ An overview of the number of interactions (Figure 8C) and the strength of interactions (Figure 8D) revealed frequent communication between tumor Scissor_Pos, myeloid Scissor_Pos, endothelial Scissor_Pos cells, and pericytes. These prognostically unfavorable cell populations, along with pericytes, formed a highly interactive hub within the TME. A chord diagram further detailed the specific ligand-receptor pairs mediating these interactions (Figure 8E), highlighting a multitude of signaling pathways involved.

Delving deeper into specific signaling networks known to be critical in aggressive GBM biology, we found that the collagen signaling network was highly active, with pericytes and endothelial Scissor_Pos cells acting as prominent senders, and various cell types, including themselves, as receivers (Figure 8F). This suggests active collagen deposition and ECM remodeling is highly related to tumor vasculature and orchestrated by these detrimental cell states. Even more strikingly, the SPP1 (osteopontin) signaling network showed exceptionally strong communication, particularly from myeloid Scissor_Pos cells (and to a lesser extent tumor Scissor_Pos and endothelial Scissor_Pos) to multiple receiver cell types, including endothelial cells and tumor cells (Figure 8G). SPP1 is a well-known matricellular protein implicated in promoting angiogenesis, inflammation, EMT, and immune suppression in various cancers, including GBM.^18^ These findings collectively suggest that the Scissor-Pos cells not only possess intrinsically aggressive functional states (mesenchymal tumor cells, M2-like myeloid cells) but also actively communicate with each other and with pericytes via ECM-remodeling and pro-angiogenic signals like SPP1, thereby cooperatively driving tumor progression.

### Spatial co-localization of ECMSig, hypoxia, Scissor-Positive cells, and pericytes in the GBM tumor microenvironment

**Figure 9:**
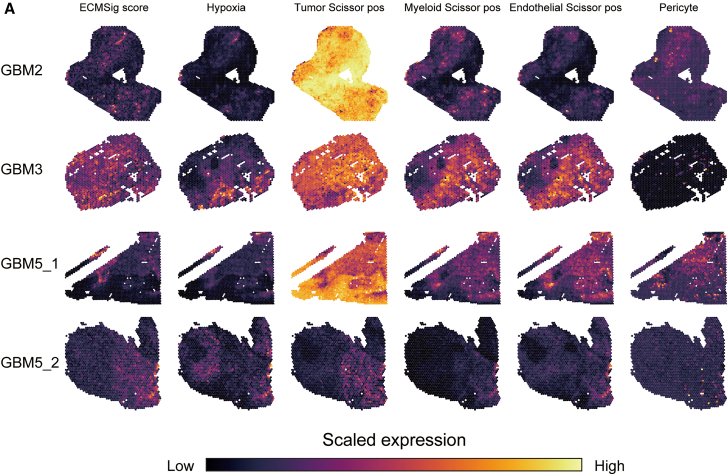
Spatial transcriptomic analysis revealing co-localization of ECMSig, hypoxia, Scissor-Positive cells, and pericytes in GBM (A) Spatial feature plots for four GBM samples. Each row represents a sample. Columns show spatial heatmaps of: ECMSig score, hypoxia signature score, tumor Scissor_Pos signature score, myeloid Scissor_Pos signature score, endothelial Scissor Pos signature score, and pericyte marker signature score. Color scale indicates scaled expression or score (low to high). Each dot represents a spatial barcoded spot.

To understand the spatial organization of these prognostically relevant cellular and molecular features within the intact tumor architecture, we leveraged publicly available spatial transcriptomics data from four GBM samples. We mapped the ECMSig score, a hypoxia gene signature score, the inferred abundance of tumor Scissor_Pos, myeloid Scissor_Pos, endothelial Scissor_Pos cells (using signature scores derived from scRNA-seq), and a pericyte marker signature across the spatial coordinates of each tumor section (Figure 9A).

The spatial heatmaps revealed striking patterns of co-localization of above signatures. Regions with high ECMSig scores consistently overlapped with areas exhibiting high hypoxia scores across all GBM samples. This spatial correlation reinforces the strong link between our ECM-related signature and tumor hypoxia. Furthermore, the inferred tumor Scissor_Pos cell populations frequently localized to these ECMSig-high and hypoxic regions. Similarly, myeloid Scissor_Pos cells and endothelial Scissor_Pos cells also showed preferential enrichment in these same microdomains. Pericyte markers, indicative of vasculature, were also often found in proximity to or overlapping with these areas of high ECMSig, hypoxia, and Scissor-Pos cell activity. This spatial analysis strongly suggests the existence of specific TME niches, likely perivascular, where high ECMSig activity, hypoxia, prognostically unfavorable Scissor-Pos tumor, myeloid, and endothelial cells, and pericytes converge. Such niches are hypothesized to be critical hubs for ECM remodeling, fostering an immunosuppressive and pro-angiogenic environment, promoting tumor cell aggressiveness, and ultimately contributing to the poor prognosis associated with a high ECMSig score. The observed co-localization provides a spatial context for the functional interactions and metabolic adaptations previously identified.

### Predicting drug sensitivity based on ECMSig stratification

**Figure 10:**
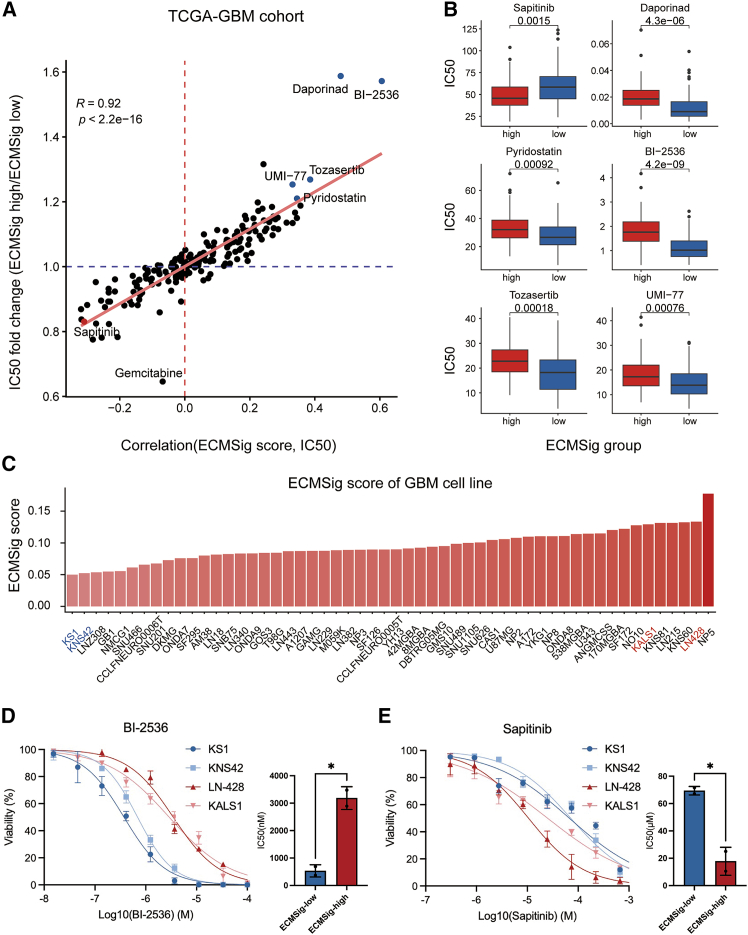
Prediction of drug sensitivity based on ECMSig stratification in TCGA-GBM (A) Scatterplot showing the relationship between the Spearman correlation of ECMSig score with predicted drug IC50 (x axis) and the log2 fold change of IC50 (ECMSig high/ECMSig low) (y axis) for a library of compounds. Red line represents linear regression fit. Dashed lines indicate zero correlation and 0-fold change. Selected drugs are labeled. Spearman correlation test. (B) Boxplots comparing predicted IC50 values for selected drugs between ECMSig-high (blue) and ECMSig-low (red) groups. q values are shown. Wilcoxon signed-rank test. Benjamini-Hochberg adjusted. (C) ECMSig scores of 54 GBM cell lines. (D) IC_50_ of BI-2536 in four GBM cell lines. (E) IC_50_ of sapitinib in four GBM cell lines.

Finally, to explore potential therapeutic strategies tailored to the distinct biological states defined by our ECMSig, we employed the oncoPredict^19^ algorithm to predict the sensitivity (IC_50_ values) of TCGA-GBM samples to a library of anti-cancer drugs based on their transcriptomic profiles. We first investigated the overall relationship between the ECMSig score and drug sensitivity (Figure 10A), identifying a strong correlation between ECMSig score and predicted drug sensitivity profiles (R = 0.92). Drugs plotted in the lower left quadrant (IC_50_ negative correlation with ECMSig score and lower IC_50_ in ECMSig-high group) are predicted to be more effective in ECMSig-high patients, while those in the upper right quadrant might be more effective in ECMSig-low patients. Comparison of predicted IC_50_ values between the two groups identified five drugs and one drug that might be more effective in ECMSig-low and ECMSig-high groups, respectively (Figure 10B).

To experimentally validate the drug-ECMSig association, we analyzed DepMap expression profiles for 54 GBM cell lines to compute ECMSig scores.^20^ We selected two ECMSig-high cell lines (LN-428, KALS1) and two ECMSig-low cell lines (KS1, KNS42) for *in vitro* assays. Consistent with our predictions, BI-2536 exhibited lower IC_50_ values in ECMSig-low cell lines, whereas sapitinib showed lower IC_50_ values in ECMSig-high cell lines (Figures 10C–10E). These results provide functional support for our computational predictions.

These findings suggest that patients stratified by the ECMSig may benefit from distinct therapeutic approaches. These computational predictions provide a valuable starting point for prioritizing drugs for further preclinical and clinical evaluation in ECMSig-defined GBM patient subgroups.

## Discussion

GBM’s aggressive nature and profound intra- and inter-tumoral heterogeneity pose significant challenges for accurate prognostication and effective treatment. In this study, we successfully developed and comprehensively validated a seven-gene ECMSig that robustly stratifies GBM patients into distinct risk groups. The ECMSig demonstrated significant and independent prognostic value for OS in the TCGA discovery cohort and was successfully replicated in two large external CGGA validation cohorts, highlighting its generalizability and potential clinical utility. The development of a nomogram integrating ECMSig with clinical variables further enhances its applicability for individualized risk assessment.

While the poor prognostic impact of ECM remodeling in GBM has been well documented, our ECMSig score provided additional biological insights for GBM. This bulk RNA-seq data-derived quantitative ECMSig score is applicable across multiple data modalities, including bulk RNA-seq, proteomics, single-cell RNA-seq, and spatial transcriptomics, enabling cross-platform assessment of ECM remodeling activity. Using multi-omics analysis, we uncovered biological associations between ECMSig levels and specific genomic alterations, distinct immune microenvironment profiles, and the predominant cellular sources driving ECM remodeling within GBM tissue.

Our multi-omics investigation revealed that the ECMSig captured a spectrum of aggressive biological features intrinsic to high-risk GBM. Genomically, while common GBM alterations like *PTEN* and *EGFR* mutations were prevalent in both risk groups, ECMSig-high tumors notably exhibited a lower frequency of *TP53* mutations and the recurrent mutation of PI3K pathway. This aligns with studies suggesting that different molecular subtypes of GBM, which may correlate with *TP53* status, have distinct prognoses and TME compositions.^9^ Transcriptomically and proteomically, ECMSig-high tumors were characterized by the activation of critical oncogenic pathways, including EMT, hypoxia, angiogenesis, and inflammatory signaling. These pathways are well-established drivers of GBM malignancy, contributing to invasion, therapeutic resistance, and immune evasion.^21^^,^^22^ The consistent enrichment of these features across both TCGA (transcriptome) and CPTAC (proteome) cohorts strengthens the biological relevance of the ECMSig.

A key finding of our study is the strong association between a high ECMSig score and a tumor-promoting, immunosuppressive microenvironment. ECMSig-high tumors displayed increased infiltration of M2-polarized macrophages, coupled with elevated hypoxia scores. Tumor-associated macrophages (TAMs), particularly the M2 phenotype, are known to foster GBM progression by promoting angiogenesis, matrix remodeling, and suppressing anti-tumor immunity.^23^^,^^24^^,^^25^ Hypoxia, a common feature in GBM, further drives TAM polarization toward an M2-like state and stimulates ECM production and remodeling, creating a vicious cycle that supports tumor growth and therapeutic resistance. The ECMSig appears to effectively capture this complex interplay between ECM dysregulation, hypoxia, and myeloid cell skewing.

Leveraging scRNA-seq data with the Scissor algorithm provided unprecedented cellular resolution into the prognostic significance of ECMSig. We identified specific Scissor-Positive tumor, myeloid, and endothelial cell subpopulations that were strongly associated with poor patient outcomes and, critically, exhibited significantly higher ECMSig scores. Functionally, tumor Scissor_Pos cells displayed a pronounced mesenchymal phenotype, a glioma cell state linked to heightened invasiveness and radioresistance. Myeloid Scissor_Pos cells were characterized by strong M2 macrophage and pro-angiogenic signatures. This suggests that the ECMSig reflects the activity of these particularly aggressive cellular states within their respective lineages. Further exploration revealed distinct metabolic reprogramming in these Scissor-Pos cells, including enhanced glycolysis and altered drug metabolism pathways, likely contributing to their aggressive phenotype and adaptation to the hypoxic TME.

The intricate communication networks within the TME are crucial for tumor progression. Our analysis highlighted robust intercellular signaling, particularly via collagen and SPP1 pathways, between these prognostically unfavorable Scissor-Pos cells (tumor, myeloid, and endothelial) and pericytes. SPP1 (osteopontin), a key component of our ECMSig, is a matricellular protein known to promote GBM angiogenesis, inflammation, EMT, and chemoresistance. The prominent SPP1 signaling, especially from myeloid Scissor_Pos cells, suggests a mechanism by which these cells actively shape the TME. Our spatial transcriptomic analysis provided a compelling visual confirmation of these interactions, demonstrating the co-localization of high ECMSig scores, hypoxia, Scissor-Pos cell signatures, and pericytes within specific tumor regions. This suggests the formation of perivascular niches where these detrimental cellular and molecular features converge to create a highly pro-tumorigenic and ECM-rich microenvironment.

From a therapeutic perspective, the ECMSig holds promise for guiding treatment decisions. Our computational drug sensitivity analysis using oncoPredict indicated that ECMSig-stratified patients might respond differently to various anti-cancer agents. For instance, based on the aforementioned observation of high TP53 mutation frequency, enrichment of cell cycle-related pathways, and downregulation of apoptosis pathways, the ECMSig-low group exhibited an increased dependency on proliferative and anti-apoptotic signaling, consistent with their sensitivity to PLK1 inhibition (BI-2536),^26^ Aurora kinases inhibition (tozasertib),^27^ and MCL-1 inhibition (UMI-77).^28^ Similarly, the NAMPT inhibitor daporinad might deplete cellular NAD^+^ pools, thereby disrupting metabolic homeostasis and indirectly impairing NAD^+^-dependent DNA repair pathways to impair ECMSig low tumor’s capacity to survive under the increase replication stress and DNA damage burden.^29^ Additionally, superior-survival genes are associated with “translation initiation,” therefore, the ECMSig low group might be sensitive to the transcriptional regulation inhibitor pyridostatin.^30^ For the ECMSig high group, these tumors display upregulated ECM remodeling and signaling pathways, which may enhance growth factor receptor activation and make them more susceptible to ERBB pathway inhibitor sapitinib.^31^

In conclusion, we have developed and validated a robust seven-gene ECM-related signature, ECMSig, which serves as an independent prognostic biomarker for GBM patients. The ECMSig captures a confluence of aggressive genomic, transcriptomic, proteomic, and cellular features, particularly highlighting the roles of hypoxia, mesenchymal transition, M2 macrophages, and specific prognostically detrimental cell states within the TME. The spatial co-localization of these elements within perivascular niches underscores the organized nature of this aggressive ecosystem. Our findings not only enhance the prognostic toolkit for GBM but also provide a deeper understanding of the ECM’s multifaceted role in tumor progression, offering potential avenues for developing personalized therapeutic interventions targeting the unique biology of ECMSig-defined patient subgroups.

### Limitations of the study

Our study has several limitations. First, ECMSig was developed on bulk RNA-seq data from TCGA-GBM and, despite validation across independent bulk, proteomic, single-cell, and spatial transcriptomic datasets, it has yet to be tested in large prospective clinical cohorts. Second, the drug sensitivity predictions were derived from pharmacogenomic modeling and experimentally validated in a limited panel of GBM cell lines; further validation in orthotopic models and patient-derived samples is warranted. Third, while analysis identified poor-prognosis cell populations with high ECMSig scores, single-cell-specific technical biases and platform differences may affect the robustness of this integration. Fourth, ECM proteins are technically challenging to quantify in proteomics due to their extracellular localization and physicochemical properties, although all components of ECMSig were detected in CPTAC data. Finally, the multi-omics associations we report are correlative and require additional functional studies to establish causality.

## Resource availability

### Lead contact

Requests for further information and resources should be directed to and will be fulfilled by the lead contact, Manqing Cao (caomanqing@tjmuch.com).

### Materials availability

There are no additional data, software, databases, or applications/tools available beyond those disclosed in the current study. All data are included in the article and supplementary data section.

### Data and code availability

• The bulk RNA sequencing (RNA-seq) data and matched clinical information can be accessed through the UCSC Xena website^32^ (GDC TCGA-GBM cohort, TPM) and the Chinese Glioma Genome Atlas (mRNA_693 and mRNA_325 cohorts).^33^^,^^34^ The genomic data of TCGA GBM cohort was available at UCSC Xena website. The GBM proteomic dataset and paired clinical information were accessed at the Clinical Proteomic Tumor Analysis Consortium (CPTAC).^35^ The single-cell transcriptomic sequencing dataset utilizing technology from the 10X Genomics platform was available under the accession number GEO: GSE182109 at the Gene Expression Omnibus (GEO) repository.^36^ The spatial transcriptomic sequencing dataset using the 10X Genomics Visium platform was under accession GEO: GSE194329.^37^ The RNA expression data of GBM cell lines were downloaded from DEPMAP website.^20^
• All analysis scripts, custom functions, and visualization code publicly available at zenodo: https://doi.org/10.5281/zenodo.17669213.
• Any additional information required to reanalyze the data reported in this article is available from the lead contact upon request.

## Acknowledgments

This work was supported by grants from the 10.13039/501100001809National Natural Science Foundation of China (no. 82403722), Postdoctoral Innovation Talent Support Program (BX20240026), and 10.13039/501100002858China Postdoctoral Science Foundation (2024M762383).

## Author contributions

Z.Z., H.X., and H.Z. contributed to the conceptualization, experimental design, functional experiments, and data analysis of the study. M.C., M.F., and Y.Y. were responsible for drafting the manuscript and revising it critically for important intellectual content. Z.P. participated in data acquisition and partial data analysis. M.C. provided final approval of the manuscript for submission.

## Declaration of interests

The authors declare that they have no competing interest for this work.

## Declaration of generative AI and AI-assisted technologies in the writing process

During the preparation of this work, the authors used ChatGPT (OpenAI) to improve the English language and readability of the text. After using this tool, the authors reviewed and edited the content as needed and take full responsibility for the content of the publication.

## STAR★Methods

### Key resources table

**Table:** 

REAGENT or RESOURCE	SOURCE	IDENTIFIER
**Chemicals, peptides, and recombinant proteins**		

BI2536	MCE	Cat# HY-50698
Sapitinib	MCE	Cat# HY-13050

**Deposited data**		

TCGA-GBM bulk RNA-seq, WES data and clinical information	UCSC Xena^32^	https://xena.ucsc.edu/
CGGA bulk RNA-seq data and clinical information	Zhao et al.^33^	https://www.cgga.org.cn/
CPTAC data of GBM	Thangudu et al.^35^	https://proteomic.datacommons.cancer.gov/pdc/
scRNA-seq data of GBM	Abdelfattah et al.^36^	GEO: GSE182109
Spatial transcriptomic data of GBM	Ren et al.^37^	GEO: GSE194329
Code used for analysis	This paper	zenodo: https://doi.org/10.5281/zenodo.17669213
DEPMAP RNA expression of GBM cell lines	Arafeh et al.^20^	https://depmap.org/portal/

**Experimental models: Cell lines**		

KS1	American Type Culture Collection (ATCC)	RRID: CVCL_1343
KNS42	American Type Culture Collection (ATCC)	RRID: CVCL_0378
LN-428	BioVector NTCC	RRID: CVCL_3959
KALS1	American Type Culture Collection (ATCC)	RRID: CVCL_1323

**Software and algorithms**		

survival v3.8.3	N/A	https://cran.r-project.org/web/packages/survival/index.html
clusterProfiler v4.14.6	Xu et al.^38^	https://bioconductor.org/packages/release/bioc/html/clusterProfiler.html
rms v8.0.0	N/A	https://cran.r-project.org/web/packages/rms/index.html
TIMER2.0	Li et al.^39^	https://compbio.cn/timer2/
maftools v2.22.0	Mayakonda et al.^40^	https://github.com/PoisonAlien/maftools
Scissor v2.0.0	Sun et al.^13^	https://github.com/sunduanchen/Scissor
CellChat v2.1.0	Jin et al.^17^	https://github.com/jinworks/CellChat
scMetabolism v0.2.1	Wu et al.^41^	https://github.com/wu-yc/scMetabolism
squidpy v1.5.0	Giovanni et al.^42^	https://squidpy.readthedocs.io/en/stable/
oncoPredict v1.2	Maeser et al.^19^	https://github.com/HuangLabUMN/oncoPredict
GraphPad Prism	GraphPad Software	https://www.graphpad.com/scientific-software/prism/

### Experimental model and study participant details

KS1 (RRID: CVCL_1343, human glioblastoma cell line from a 45 year old woman) and KNS42 (RRID: CVCL_0378, human glioblastoma cell line from a 16 year old man) cells were cultured in Dulbecco’s Modified Eagle Medium/Nutrient Mixture F-12 (DMEM/F12; 10-092-CVRC) supplemented with 10% heat-inactivated fetal bovine serum and 1% penicillin–streptomycin. LN-428 cells (RRID: CVCL_3959, human glioblastoma cell line from a 48 year old man) were maintained in high-glucose DMEM (DMEM: 10-013-CVRC) containing 10% heat-inactivated FBS and 1% P/S. KALS1 cells (RRID: CVCL_1323, human glioblastoma cell line from a 74 year old woman) were cultured in RPMI-1640 (10-040-CVRC) medium supplemented with 10% heat-inactivated FBS and 1% P/S. All cell lines were tested for mycoplasma contamination and were grown at 37°C in a humidified incubator with 5% CO_2_.

### Method details

#### Data acquisition

For this research, the bulk RNA sequencing (RNA-seq) data and matched clinical information was collected from the UCSC Xena website^32^ (GDC TCGA-GBM cohort, TPM) and the Chinese Glioma Genome Atlas (mRNA_693 and mRNA_325 cohorts).^33^^,^^34^ Only cases diagnosed as primary GBM (sample barcode ends with “01A”) were included; recurrent GBM cases were excluded from all analyses. The genomic data of TCGA GBM cohort was downloaded from UCSC Xena website. The GBM proteomic dataset and paired clinical information were collected from the Clinical Proteomic Tumor Analysis Consortium (CPTAC, https://proteomics.cancer.gov/programs/cptac).^35^ The single cell transcriptomic sequencing dataset was downloaded from GEO: GSE182109 at the Gene Expression Omnibus (GEO) repository.^36^ The spatial transcriptomic sequencing dataset was obtained from GEO: GSE194329.^37^ The RNA expression matrix of GBM cell lines were downloaded from DEPMAP.^20^

#### Identification of differentially expressed and prognosis-related genes

The differentially expressed genes between GBM tumor samples and normal tissue samples were obtained from the Gene Expression Profiling Interactive Analysis website (GEPIA2).^43^ Genes with log2(Foldchange) ≥ 1 and adjusted p value <0.05 were determined as upregulated genes. To identify the genes related to poor survival of GBM patients, survival analysis of each gene on the TCGA-GBM cohort was conducted using the Survival and Survminer packages. The median expression value was used to stratify patients into two distinct groups (high or low). The ‘survfit’ function was employed for the construction of Kaplan-Meier survival curves and the ‘ggsurvplot’ function was used to plot it.

#### Pathway enrichment analysis and signature analysis

Pathway enrichment analysis was performed using the clusterProfiler package.^38^ The gene sets of HALLMARK, Gene Ontology (GO), Kyoto Encyclopedia of Genes and Genomes (KEGG), and Reactome pathways were obtained from the Molecular Signatures Database (MSigDB).^44^ Signature scores were calculated using Gene Set Enrichment Analysis (GSEA) or single-sample GSEA. Immune deconvolution result of TCGA-GBM cohort was downloaded from the TIMER2 website.^39^

#### Construction and validation of the prognostic signature

To construct the prognostic signature, we first curated a list of candidate genes. This involved identifying the intersection of three gene sets derived from the TCGA-GBM cohort: (1) genes upregulated in GBM tumors compared to adjacent normal tissues; (2) genes significantly associated with poor OS; (3) genes belonging to these GO pathways: including “GOBP positive regulation of cell adhesion”, “GOCC collagen containing extracellular matrix”, “GOMF collagen binding”, “GOMF extracellular matrix structural constituent”, “GOMF fibronectin binding”. The expression matrix of these intersected candidate genes and the clinical information was used as input for a Least Absolute Shrinkage and Selection Operator (LASSO) Cox proportional hazards regression model. Genes with non-zero coefficients at this optimal minimal λ were incorporated into the final signature. The multivariate survival analysis was performed with the ‘coxph’ function and the nomogram was established using the rms package. The prognostic value of this signature was validated in the CGGA 693 cohort and CCGA 325 cohort with the same methods.

#### Genomic alteration analysis

Genomic alteration analysis was performed on TCGA GBM samples for which both somatic mutation data and corresponding transcriptome data were available. Somatic mutation data were processed and visualized using the maftools package.^40^ Mutations (MAF file) of high/low score groups were loaded by maftools and used to: (1) identification and visualization of frequently mutated genes within each group; (2) visualization of frequently mutated oncogenic pathways; (3) differential mutation analysis between two groups; (4) assessment of co-occurrence and mutually exclusive mutation patterns among highly mutated genes; (5) exploration of potential drug-gene interactions based on the identified genomic variants.

#### Proteomic analysis

Unshared log ratio of proteins in GBM samples were acquired from the Clinical Proteomic Tumor Analysis Consortium (CPTAC) database (GBM Confirmatory cohort). Only samples with both available proteomic profiles and corresponding clinical data, including survival information, were included in this analysis. For each selected sample, the previously established prognostic signature score was calculated using their respective protein expression levels. Patients were then dichotomized into high- and low-risk groups based on the median signature score. Differential protein expression analysis was conducted using the Wilcoxon rank-sum test.

#### Single-cell RNA-seq analysis

Preprocessing of the scRNA-seq data, including quality control, normalization, and clustering, was performed as previously described. This process identified eight primary cell types, including glioma cells, endothelial cells, myeloid cells, pericytes, oligo cells, B cells, T cells, and other cells. Scissor^13^ was employed to identify prognostically relevant cell subsets within glioma, myeloid cells, endothelial cells, and pericytes. The single-cell expression matrices for these cell populations were individually processed. For each cell type, the scRNA-seq matrix, along with the bulk RNA-seq (TPM expression) data and corresponding patient survival information of TCGA-GBM cohort were used as input. Using Scissor’s Cox regression mode, cells significantly associated with GBM patient mortality were identified. Cells positively correlated with mortality (poor prognosis) were designated as “Scissor-positive” (Scissor+), while those negatively correlated (favorable prognosis) were termed “Scissor-negative” (Scissor-). To characterize the phenotypic features of these cell subsets, further analyses were conducted using scanpy.^45^ Differentially expressed genes of each subset were identified and several signature scores were calculated, including the prognostic survival signature score, previously well-defined tumor cell states,^14^ and myeloid cell functional signatures.^15^ Metabolic reprogramming within these Scissor-identified cell subsets was investigated using the scMetabolism package.^41^ Intercellular communication between Scissor-identified cell subsets and other TME cell types was inferred using CellChat.^17^

#### Spatial transcriptomics analysis

Spatial transcriptomics data of 4 GBM samples were utilized for this study. Preprocessing of the spatial transcriptomics data was performed following previously established protocols. The top 200 most highly expressed genes (marker genes) characterizing the “Scissor-positive” (Scissor+) cell subset were used to define a signature score. This score was calculated for each spatial spot to infer the enrichment and localization of Scissor+ like cells across the tissue sections. Similarly, to visualize the spatial distribution of blood vessels, a signature score was derived using the top 200 most highly expressed genes characteristic of pericytes. Furthermore, to assess regional hypoxia within the tumor microenvironment, the HALLMARK_HYPOXIA gene set from MSigDB was employed. All spatial transcriptomic analyses were conducted using the squidpy package.^42^

#### Drug sensitivity prediction

To explore potential therapeutic vulnerabilities associated with the prognostic survival signature, we performed in silico drug sensitivity prediction using gene expression data. Transcriptomic data (RNA-seq TPM values) from The Cancer Genome Atlas Glioblastoma Multiforme (TCGA-GBM) cohort were utilized. Patients within the TCGA-GBM cohort were first stratified into high-risk and low-risk groups based on their calculated survival signature scores, as previously described. Drug sensitivity prediction was then conducted for each sample using the oncoPredict R package.^19^ The oncoPredict analysis was run using the GDSC2 pre-trained model.

#### Determination of IC_50_ values

KS1, KNS42, LN-428, and KALS1 cell lines were cultured under standard growth conditions and seeded into 96-well plates at a density of 1000 cells/well. After an overnight attachment period, cells were treated with serial dilutions of BI2536 (MCE, Cat#HY-50698) or Sapitinib (MCE, Cat#HY-13050), prepared in cell culture medium. Following 72 h incubation, cell viability was assessed using a CellTiter-Glo luminescent cell viability assay according to the manufacturer’s instructions. Viability (%) was calculated relative to untreated controls. The resulting dose–response data were plotted as the percentage of viable cells versus the log_10_ concentration of BI2536 or Sapitinib. IC_50_ values were obtained by fitting the data to a variable slope logistic model using GraphPad Prism version 10 (GraphPad Software, San Diego, CA). The IC_50_ is: mean ± standard error.

### Quantification and statistical analysis

All statistical analyses and plots were conducted using R (v4.4.1) and Python (v3.10.16). The log-rank test was employed for Kaplan-Meier survival analysis, while the Spearman correlation coefficient was used to evaluate linear relationships. Wilcoxon test was performed for multiple comparisons. Benjamini-Hochberg adjusted P-value was calculated when multiple testing is performed. P-values/adjusted p-values were indicated within the plots to denote statistical significance (∗P <0.05, ∗∗P <0.01, ∗∗∗P <0.001, ∗∗∗∗P <0.0001, ns: nonsignificant).

## Supplemental information

Document S1. Figures S1–S3 and Table S1

Table S2. IC_50_ of BI-2536 and Sapitinib in four GBM cell lines
