# Supplementary material for: Multi-omics profiling-derived signature links cellular ecosystem to glioblastoma prognosis
Source: iScience. 2026 May 18;29(6):115982. doi: 10.1016/j.isci.2026.115982 (PMC13197639; doi:10.1016/j.isci.2026.115982)
Supplement: Document S1. Figures S1–S3 and Table S1 [file mmc1.pdf]

iScience, Volume *29*

Supplemental information

Multi-omics profiling-derived signature links cellular ecosystem to glioblastoma prognosis

Zhen Zhang, Hao Xu, Haijing Zheng, Zhaolong Pan, Mei Feng, Yongchang Yang, and Manqing Cao

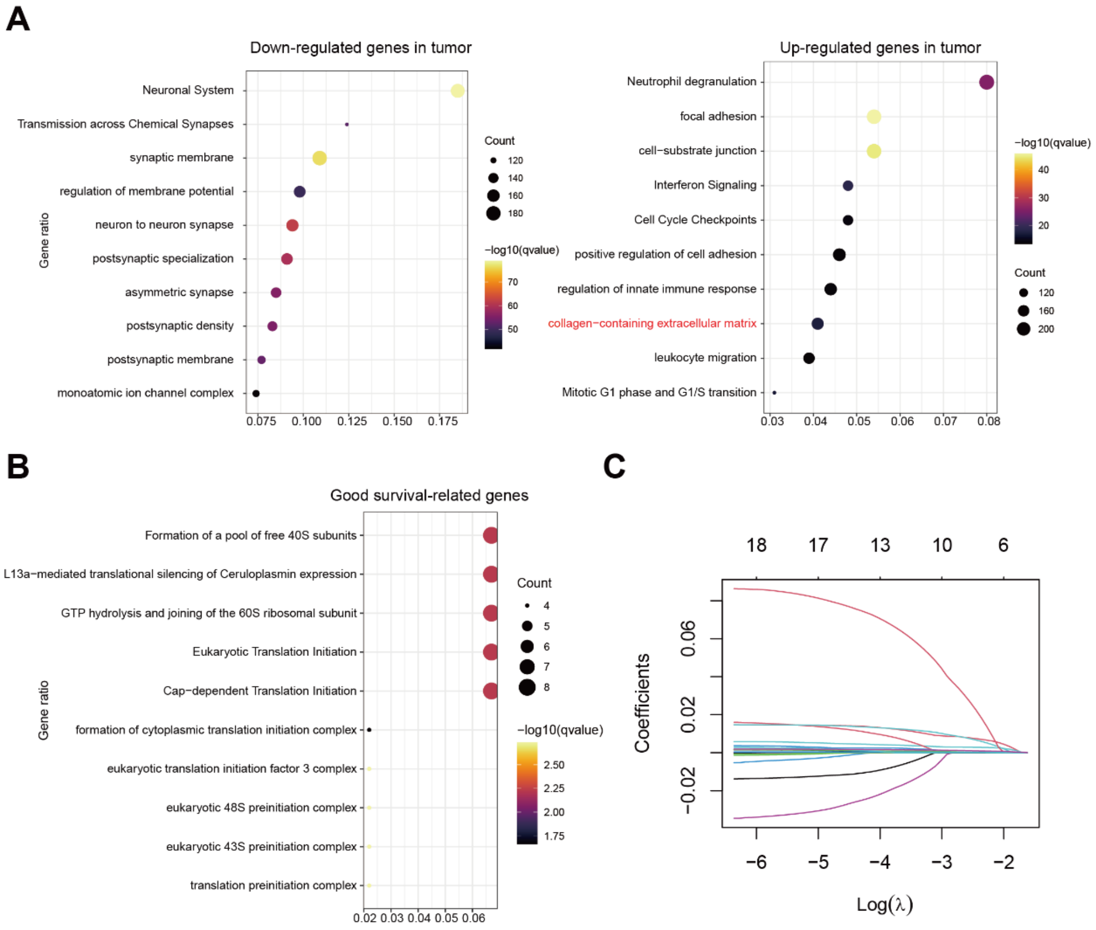

Figure S1. Functional enrichment analysis of gene sets in GBM. Related to Figure 1.

A. Gene Ontology (GO) enrichment analysis of differentially expressed genes in GBM compared to normal tissue. Dot plot displaying the top enriched GO biological process terms. The x-axis represents the gene ratio. The size of the dots corresponds to the number of genes associated with each term, and the color intensity reflects the statistical significance (-log10(q-value)). Benjamini-Hochberg adjusted.

B. GO enrichment analysis of genes associated with good overall survival (OS) in GBM patients. Benjamini-Hochberg adjusted.

C. LASSO coefficient profiles of the 19 candidate prognostic genes. Each colored line represents a gene. The x-axis shows the log(λ) value (penalization parameter), and the y-axis shows the regression coefficients.

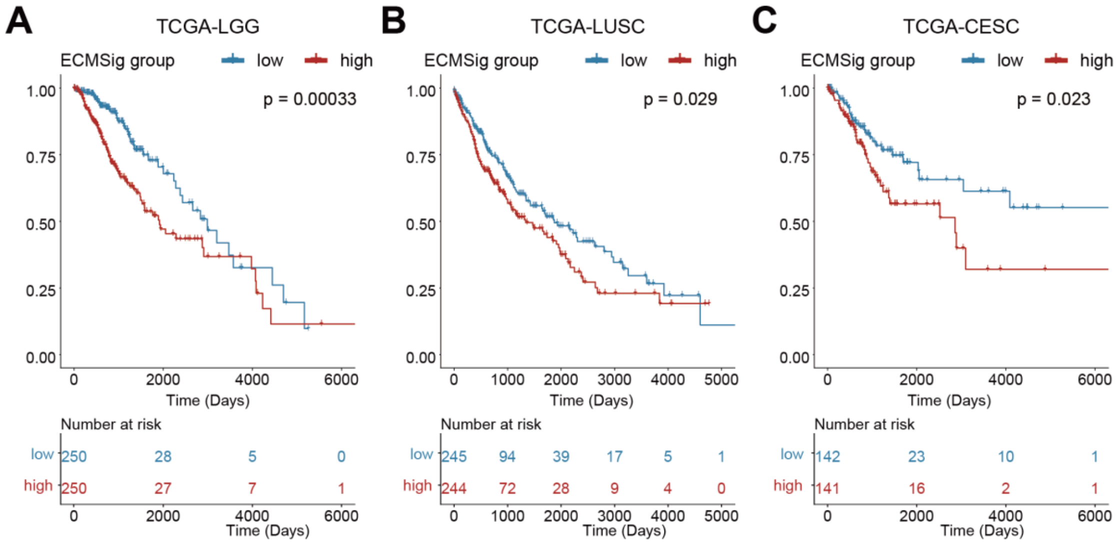

Figure S2. Prognostic value of ECMSig score in TCGA cohorts. Related to Figure 2.

A-C. Survival analysis of ECMSig score in TCGA-LGG **(A**, low-grade glioma), TCGA-LUSC (**B**, lung squamous cell carcinoma), and TCGA-CESC (**C**, cervical squamous cell carcinoma and endocervical adenocarcinoma) cohorts.

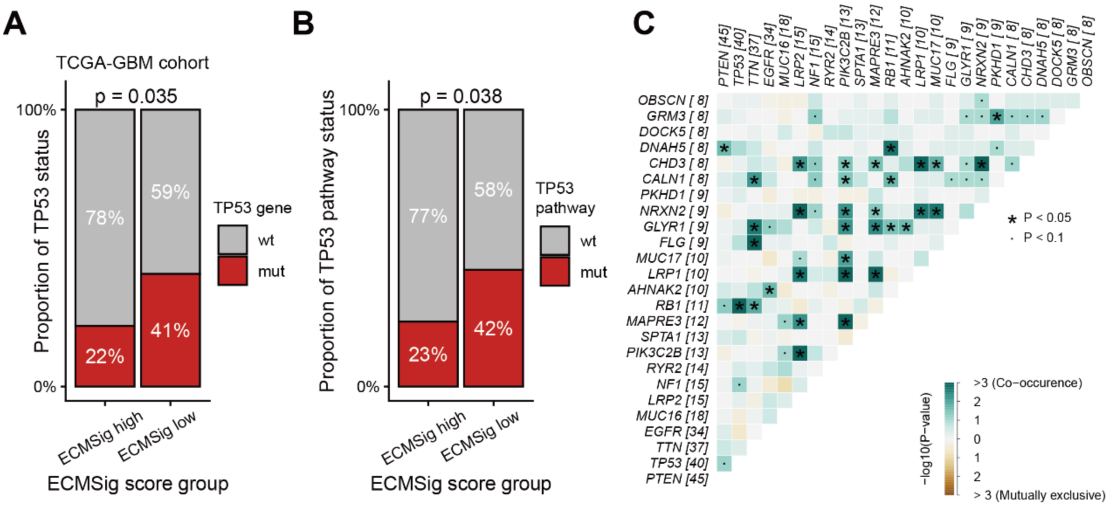

Figure S3. Mutational landscape and co-occurrence patterns in the TCGA-GBM cohort. Related to Figure 3.

A-B. Mutation frequencies of *TP53* gene (**A**) and TP53 pathway (**B**) in the TCGA-GBM cohort, stratified by ECMSig status (low vs. high). Bars represent the proportion of mutated and wildtype samples within each group. Numbers above bars indicate the percentage of mutated samples versus the total samples in that group. Asterisks denote statistical significance of the difference in mutation frequency between ECMSig low and high groups (*P < 0.05, **P < 0.01). Fisher's exact test.

C. Heatmap illustrating pairwise co-occurrence and mutual exclusivity of mutations among frequently mutated genes in the TCGA-GBM cohort. Gene names are listed on the axes, with numbers in brackets indicating mutation counts for each gene. The color intensity of each cell represents the statistical significance (-log10(P-value)) of the association, with specific colors indicating co-occurrence (green) or mutual exclusivity (brown). Asterisks indicate significance levels (*P < 0.05, ·P < 0.1).

Table S1. Primary targets and biological rationale of 6 candidate drugs for GBM.

Drug	Main known target(s)	Sensitive in group	Biological rationale
BI-2536	PLK1 (Polo-like kinase 1) and BRD4	ECMSig low	Cell cycle regulation related to higher mutation rate of TP53 and "E2F/G2M checkpoint" pathway enriched in ECMSig low group
Daporinad	NAMPT (Nicotinamide phosphoribosyltransferase)	ECMSig low	NAD metabolism associated with DNA repair, sensitive in high proliferation ECMSig low group
Pyridostatin	G-quadruplex DNA structures	ECMSig low	Transcriptional regulation is related to enriched "translation initiation" pathway in good survival genes
Tozasertib	Aurora kinases (Aurora A/B/C)	ECMSig low	Mitosis regulation and cell cycle related to higher mutation rate of TP53 and "E2F/G2M checkpoint" pathway enriched in ECMSig low group
UMI-77	MCL-1 (Myeloid cell leukemia-1, anti-apoptotic BCL-2 family protein)	ECMSig low	Apoptosis pathway down-regulated in ECMSig low group
Sapitinib	ERBB family (EGFR, ERBB2, ERBB3)	ECMSig high	EGFR pathway is related to upregulated EMT in ECMSig high group
